# Risk-Taking Behaviors and Adherence to HIV Pre-Exposure Prophylaxis in Users of Geosocial Networking Apps: Real-World, Multicenter Study

**DOI:** 10.2196/22388

**Published:** 2020-10-14

**Authors:** Hongyi Wang, Jing Zhang, Zhenxing Chu, Qinghai Hu, Willa Dong, Xiaojie Huang, Yaokai Chen, Hui Wang, Xiaoqing He, Lukun Zhang, Zhili Hu, Rantong Bao, Shangcao Li, Hang Li, Sitong Cui, Xia Jin, Haibo Ding, Wenqing Geng, Yongjun Jiang, Junjie Xu, Hong Shang

**Affiliations:** 1 NHC Key Laboratory of AIDS Immunology (China Medical University), National Clinical Research Center for Laboratory Medicine, The First Affiliated Hospital of China Medical University Shenyang, Liaoning Province China; 2 Key Laboratory of AIDS Immunology, Chinese Academy of Medical Sciences Shenyang China; 3 Key Laboratory of AIDS Immunology of Liaoning Province Shenyang China; 4 Collaborative Innovation Center for Diagnosis and Treatment of Infectious Diseases Hangzhou China; 5 Department of Health Behavior, Gillings School of Global Public Health, The University of North Carolina at Chapel Hill Chapel Hill, NC United States; 6 Center for Infectious Diseases, Beijing Youan Hospital, Capital Medical University Beijing China; 7 Chongqing Public Health Medical Center Chongqing China; 8 Shenzhen Third People’s Hospital Shenzhen China

**Keywords:** men who have sex with men (MSM), MSM, HIV/AIDS prevention, Pre-exposure prophylaxis (PrEP), geosocial networking app, dating app, adherence, regimen switch

## Abstract

**Background:**

Over half of men who have sex with men (MSM) use geosocial networking (GSN) apps to encounter sex partners. GSN apps’ users have become a unique large subpopulation among MSM for interventions concerning HIV prevention and control. Pre-exposure prophylaxis (PrEP) is a promising measure for HIV prevention, especially for MSM, but its effectiveness largely depends on medication adherence. However, little is known about PrEP adherence among GSN apps’ users, which is critical to addressing the overall optimization of PrEP compliance outside of clinical trials in the context of large-scale implementation.

**Objective:**

The objective of this study is to understand the correlation between GSN apps’ use and medication adherence among MSM receiving PrEP, with the aim to increase their awareness about PrEP use in order to increase adherence.

**Methods:**

This study based on the China Real-world Oral intake of PrEP (CROPrEP) project, a multicenter, real-world study of Chinese MSM on daily and event-driven PrEP. Eligible participants completed a detailed computer-assisted self-interview on sociodemographic, GSN apps’ use, and sexual behavior. Then participants were followed up for 12 months and assessed for various characteristics (eg, PrEP delivery, adherence assessment, PrEP coverage of sexual activities, and regimens switch). A generalized estimation equation was used to analyze the predictors of medication adherence and regimen conversion among GSN apps’ users and nonusers.

**Results:**

At baseline, 756 of the 1023 eligible participants (73.90%) reported primarily using GSN apps to seek sexual partners, and GSN apps’ users are more likely to have high-risk behaviors such as multiple sex partners and condomless anal intercourse than other nonusers (all *P*<.05). During follow-up, GSN apps’ users had a significantly low level of pill-counting adherence than nonusers (adjusted odds ratio [aOR] 0.8, 95% CI 0.6-1.0, *P*=.038). In the event-driven group, GSN apps’ users had marginally lower levels of self-reported adherence (aOR 0.7, 95% CI 0.4-1.0, *P*=.060) and lower PrEP coverage of sexual practices (aOR 0.6, 95% CI 0.4-1.0, *P*=.038). Additionally, GSN apps’ users seemed more likely to switch from event-driven to daily regimen (aOR 1.8, 95% CI 0.9-3.3, *P*=.084).

**Conclusions:**

GSN apps’ users are highly prevalent among MSM, despite their higher sexual risk and lower adherence levels, suggesting that eHealth needs to be introduced to the GSN platform to promote PrEP adherence.

**Trial Registration:**

Chinese Clinical Trial Registry ChiCTR-IIN-17013762; https://tinyurl.com/yy2mhrv4.

**International Registered Report Identifier (IRRID):**

RR2-10.1186/s12879-019-4355-y

## Introduction

Men who have sex with men (MSM) are estimated to have a 20-fold higher risk of new HIV infections than the general population on average, with MSM contributing to more than half of new HIV infections in western and central Europe and North America, and 30% in Asia and the Pacific [1]. Digital technology has changed the way MSM lives and seeks sexual partners, and a meta-analysis estimated that 50%-80% of MSM reported using geosocial networking (GSN) apps for sexual encounters [2,3]. Recent studies indicate an increase in GSN app users with high-risk sexual behaviors (ie, condomless and multisex partners); however, effective interventions for HIV prevention among this group remain an underexplored topic [2-7].

Pre-exposure prophylaxis (PrEP) is a promising novel HIV prevention method, and its coverage continues to expand globally [8]. Adherence is probably the strongest determinant of eﬀectiveness for PrEP based on evidence from existing clinical trials and real-world studies [9-12], and optimizing adherence has become a common goal across various settings globally. Recent studies have found that GSN apps may facilitate the roll out of PrEP programs [13-15], though more information is needed on the role of GSN apps in PrEP initiation and adherence. In 2017, a large-scale survey of GSN apps’ users among MSM in Europe and Central Asia reported that 10% of MSM were using PrEP in the last 3 months, and 33% were planning to start PrEP in the next 6 months [16]. A study on Grindr use among MSM from the United States found that those with recent GSN apps’ use had a higher percentage of initiating PrEP compared with nonusers (24.6% vs 14%, *P*<.001) [15]. The above evidence suggests that MSM with GSN apps’ use experience may comprise a sizeable proportion of PrEP users, but we do not know exactly the size and risk level they occupy among PrEP users in China. In addition, despite the importance of adherence for PrEP effectiveness based on existing clinical trials and real-world studies among the overall MSM population, there is little information on these outcomes for MSM who are using GSN apps. In 2019, the updated WHO guidelines for PrEP recommended both daily and event-driven regimens for PrEP among MSM [17], raising concerns about regimen switches. The limited available research evidence suggests that the switch between daily and event regimens is more common when 2 regimens are available [18]. Understanding adherence and regimens switch among GSN apps’ users and addressing the barriers preventing adherence will be crucial to the long-term success of PrEP interventions in the Chinese MSM population. Discontinuation of PrEP is another concern and may hinder its prevention effect [19,20]. However, research has only scarcely explored the effect of app use behavior on PrEP discontinuation.

Therefore, this study aims to understand the behavioral characteristics of GSN apps’ users among PrEP users and assess their adherence, regimen conversion, and discontinuation in a real-world study in China with options for daily and event-driven PrEP regimens, to explore new directions for improving adherence.

## Methods

### Study Design and Participants

This study was based on the China Real-world Oral intake of PrEP (CROPrEP) project [21], a multicenter, real-world study of daily or event-driven emtricitabine/tenofovir disoproxil fumarate (TDF/FTC) PrEP regimens among HIV-negative MSM at high risk of HIV infection in China. From December 2018 to October 2019, participants were recruited from 4 cities including Shenyang (First Affiliated Hospital of China Medical University), Beijing (Beijing You An Hospital of Capital Medical University), Shenzhen (Shenzhen Third People’s Hospital), and Chongqing (Chongqing Public Health Medical Treatment Center). Eligible participants reported GSN apps’ use and sexual characteristics at baseline and then began quarterly follow-up visits (eg, free PrEP distribution, medication adherence assessments, and computer-assisted self-interview [CASI]) over 12 months. The expected follow-up time was from October 2019 to October 2020. Other methodological considerations, such as inclusion and exclusion criteria for eligible participants, are detailed in this study protocol paper [21].

### Procedures

Data analysis for this study was conducted from December 2018 to January 2020. Eligible participants completed 6 follow-up visits at baseline, 1, 3, 6, 9, and 12 months. At enrollment, after receiving professional medication counseling from doctors on daily and event-driven regimens, participants received free TDF/FTC as PrEP and chose between taking PrEP on a daily basis, or following an event-driven regimen (consisting of 2 pills 2-24 hours before sexual intercourse or 1 pill if the last medication was taken 1-6 days ago, and a pill every 24 h from the first drug intake during the period of sexual activity) [18]. Participants were permitted to change regimens during the study. A detailed computer-assisted self-administrated questionnaire on GSN apps’ use (referring to using GSN apps as the main venue for seeking sexual partners during the previous 3 months), sociodemographic information, sexual behavior, and substance use was also filled out by eligible participants to understand the distribution and characteristics of GSN apps’ users among MSM on PrEP.

At each clinical follow-up visit, participants completed assessments of adherence through pill counts and CASI. Regimen switching events were measured in the CASI. Participants were required to return the remaining pills at each visit in exchange for a dose of TDF/FTC that would cover the next visit, and the pills were counted by study staff. Participants who tested positive for HIV discontinued TDF/FTC and were offered counseling and referral services. A unique 6-digit number was assigned to participants in place of their real names to protect privacy throughout the study. For each follow-up visit, participants receive 50 RMB (about US $7.07) as compensation for travel and missed work.

### Outcomes and Core Variables

#### Primary Outcomes

In our study, the primary outcomes were pill-counting adherence and regimen switches for PrEP. Participants were asked to return surplus pills at each follow-up, and adherence was calculated by counting the number of pills actually taken and dividing by the number of theoretical pills to be taken during that period. For the event-driven group, the theoretical number of pills to be taken is calculated according to the medication regimen: 2 pills of TDF/FTC orally 2-24 hours before sex, 1 pill 24 and 48 hours after the first dose (if the interval between the last dose is within 1-6 days, take 1 pill before sex), and 1 pill every 24 hours during continuous sex. The total number of pills taken in a week does not exceed 7. The numbers of drug taking days and sex days of the drug users were collected via the questionnaire; pill-counting adherence greater than 90% was defined as an adherence to PrEP. Participants were allowed to change regimens, and the date and direction of medication regimens switching from an event-driven regimen to a daily regimen or vice versa were collected through self-administered questionnaires during follow-up.

#### Secondary Outcomes

Secondary outcomes included self-reported adherence, PrEP coverage of last sex events, and PrEP discontinuation. Self-reported adherence was assessed by a questionnaire on whether the medication was missed. We calculated recent sexual behavior with PrEP based on self-administered questionnaires and adjusted the data based on weekly records of pill taking and sexual practices. PrEP discontinuation excludes those who discontinue PrEP due to seroconversion to HIV positivity.

#### GSN Apps’ Use, Sexual Behaviors, and Other Observation Variables

The GSN app platforms mentioned in the questionnaire included *Blued, Jacked, Momo*, and other common gay social apps with geographical networking. Other adherence-related variables were also gathered via questionnaires. Specifically, questionnaires collected data on sociodemographics (eg, age, occupation, education), sexual behavior (ie, sexual role, number of sexual partners, type of sexual partners, condomless anal intercourse, and rectal douching), and substance use during the past 3 years. For sexual partners, casual partners were defined as one-night stands, as opposed to regular partners. Substance use was defined as the use of rush (poppers or alkyl nitrites), MDMA (3,4-methylenedioxymethamphetamine; ecstasy), ice, amphetamines, tramadol, or ketamine.

### Statistical Analysis

The distribution of demographic, behavioral, and PrEP-related characteristics of GSN apps’ users and nonusers among MSM on PrEP (such as sexual behavior, substance use, and the choice of regimen) was compared using chi-square analysis or Fisher exact test, as appropriate. To clarify the determinants of PrEP adherence and regimen switches, generalized estimation equation with logistic models was used for analysis to obtain robust results by controlling for the correlation between observed variables [22,23], given that adherence, PrEP coverage of last sex events, and regimen switches were evaluated at each follow-up among GSN apps’ users and nonusers. In this model, at each follow-up, data on time-dependent variables such as sexual behavior, drug use, and psychological factors were analyzed, and only variables statistically significant at the *P*<.20 level were entered into the multivariate analysis with adjustment for demographic confounding factors including ethnicity, monthly income, and residence. All analyses were performed using SPSS version 24.0 (IBM). All *P*-values and confidence intervals were 2-sided.

### Ethics Statement

This study was approved by the Institutional Review Board at the 4 research centers and research procedures were carried out strictly in accordance with relevant guidelines ([2018]2015–139-5) and the Declaration of Helsinki. Written informed consents were obtained from participants prior to starting any study procedure. This study is registered with the Chinese Clinical Trial Registry (Trial registration number ChiCTR-IIN-17013762).

## Results

### Participant Demographics

**Figure 1:**
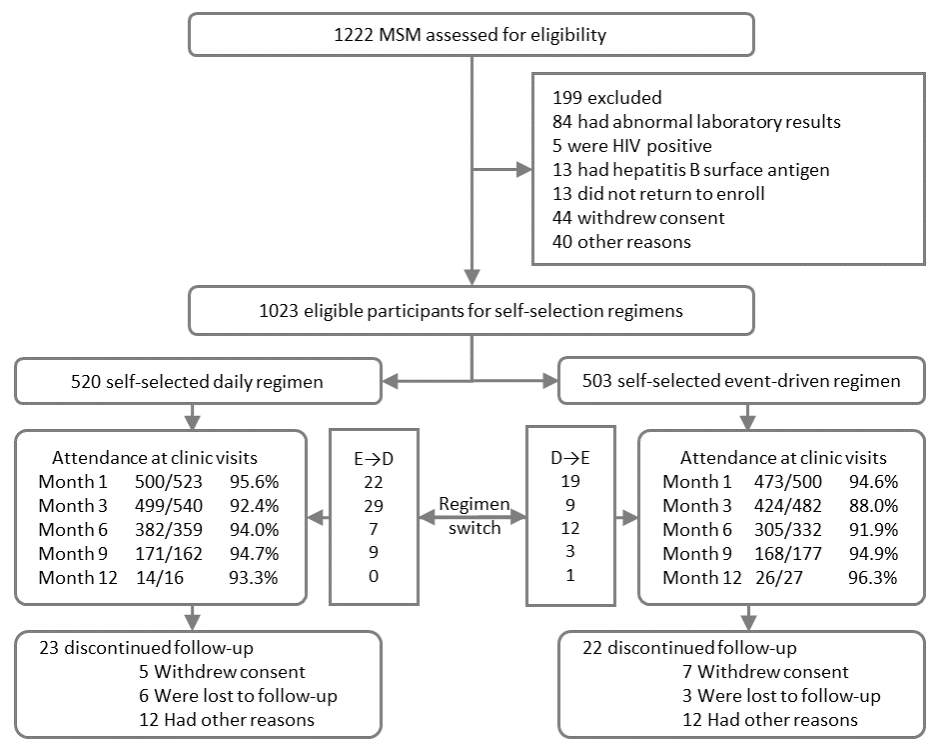
Enrollment and follow up of the study participants. D→E: switching from daily to event-driven regimen; E→D: switching from event-driven to daily regimen; MSM: men who have sex with men.

Of the 1222 MSM screened, 1023 eligible participants were enrolled, including 520 MSM who chose daily PrEP and 503 MSM who chose event-driven PrEP at baseline (Figure 1). At PrEP initiation, the median age was 29 years (IQR 25-35), 81.23% (831/1023) of participants self-reported having a university degree or above, and 54.55% (558/1023) of participants self-identified as single.

### GSN Apps’ Usage and Characteristics of GSN App Users

As much as 73.90% (756/1023) of eligible participants reported primarily using GSN apps to seek sexual partners in the past 3 months. GSN apps’ users were younger and had higher education. A detailed distribution of sociodemographic characteristics is shown in Table 1.

Participants who use GSN apps were more likely to engage in risk behaviors than those not using GSN apps. Specifically, GSN apps’ users had more male casual sexual partners (≥4: 34.9% [264/756] vs 21.7% [58/267], *P*<.001) and more condomless anal intercourse (CAI; 1-2: 19.6% [148/756] vs 14.2% [38/267], ≥3: 42.2% [319/756] vs 39.3% [105/267], *P*=.034) than nonusers. However, there was no statistical difference between GSN apps’ users and nonusers in the choice of medication regimens.

**Table 1:** Baseline characteristics of MSM on PrEP who did and did not report mainly seeking partners through geosocial networking apps during the past 3 months (N=1023).

Characteristics					GSN apps’^a^ nonuser (N=267), n (%)	GSN apps user (N=756), n (%)	χ^2^ (*df*)	*P*-value
**Age (years)**							6.33 (2)	.042
	18-24				53 (19.9)	183 (24.2)		
	25-34				128 (47.9)	387 (51.2)		
	35-65				86 (32.2)	186 (24.6)		
Han Ethnic					239 (89.5)	680 (89.9)	0.04 (1)	.840
Local residents of research centers					249 (93.3)	707 (93.5)	0.02 (1)	.883
Having a university degree or above					200 (74.9)	631 (83.5)	9.48 (1)	.002
Average annual income ≥567 (US $)					177 (66.3)	510 (67.5)	0.12 (1)	.727
**Marital status**							18.06 (1)	<.001
	Married/Cohabitation with a woman				21 (7.9)	46 (6.1)		
	Cohabitation with a man				121 (45.3)	251 (33.2)		
	Single				116 (43.4)	442 (58.5)		
	Separated, divorced, or widowed				9 (3.4)	17 (2.2)		
Ever received HIV PEP^b^ in the past 6 months					12 (4.5)	53 (7.0)	2.10 (1)	.147
**PrEP^c^ dosing regimens**							0.00 (1)	.968
	Event-driven regimen				136 (50.9)	384 (50.8)		
	Daily regimen				131 (49.1)	372 (49.2)		
Homosexual					198 (74.2)	605 (80.0)	4.03 (1)	.045
**Behaviors in the past 3 months**								
	**Sexual roles with male**						6.69 (3)	.082
				Oral	1 (0.4)	6 (0.8)		
				Top	81 (30.3)	206 (27.2)		
				Bottom	58 (21.7)	223 (29.5)		
				Versatile	127 (47.6)	321 (42.5)		
	**Number of male sex partners**						31.73 (2)	<.001
				0-1	76 (28.5)	105 (13.9)		
				2-3	86 (32.2)	246 (32.5)		
				≥4	105 (39.3)	405 (53.6)		
	**Number of male casual sex partners**						38.70 (2)	<.001
			0		130 (48.7)	213 (28.2)		
			1-3		79 (29.6)	279 (36.9)		
		≥4			58 (21.7)	264 (34.9)		
	**Number of CAI^d^**						6.79 (2)	.034
		0			124 (46.4)	289 (38.2)		
		1-2			38 (14.2)	148 (19.6)		
		≥3			105 (39.3)	319 (42.2)		
Substance use					94 (35.2)	389 (51.5)	20.90 (1)	<.001
STI^e^ symptoms					18 (6.7)	69 (9.1)	1.41 (1)	.236
Ever tested HIV in the past 12 months					245 (91.8)	718 (95.0)	3.69 (1)	.055

^a^GSN: geosocial networking.

^b^PEP: postexposure prophylaxis.

^c^PrEP: pre-exposure prophylaxis.

^d^CAI: condomless anal intercourse.

^e^STI: sexually transmitted infection.

### GSN Apps’ Use, Adherence, and Regimen Switches for PrEP

By January 2020, 97.07% (993/1023) of participants completed at least one clinical visit, with a total of 2962 follow-up visits, and the median follow-up time was 0.50 person-years, equivalent to 513.07 person-years.

**Figure 2:**
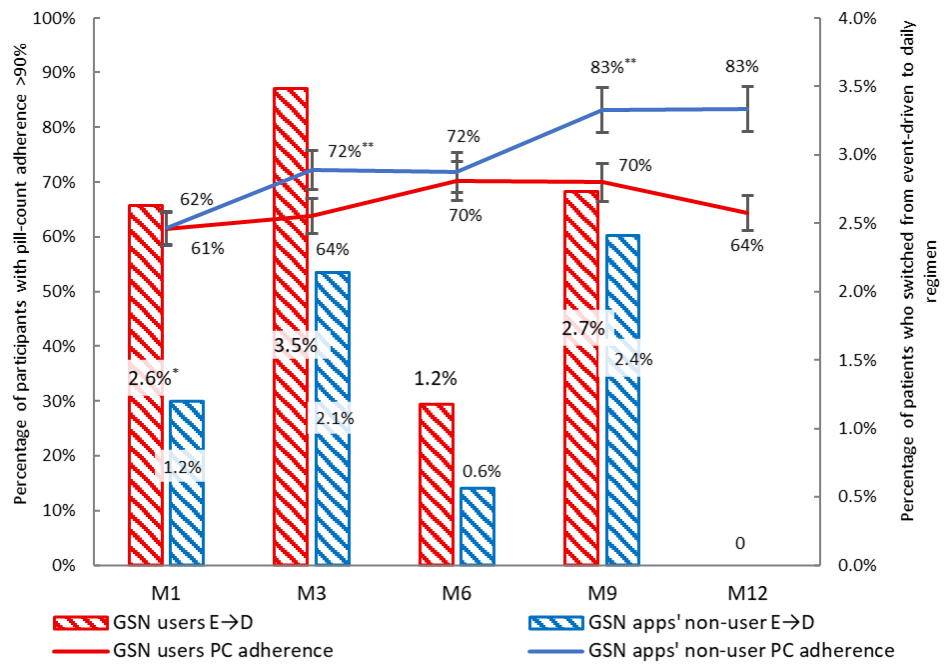
Pill-counting adherence and regimen switches for PrEP among GSN users and nonusers during follow-up The difference in the rate for pill counting adherence and regimen switching between GSN users and nonusers at each follow-up visit was marked as *P<.20, **P<.05. E→D: Switching from event-driven to daily regimen; GSN: geosocial networking; PC: pill counting; PrEP: pre-exposure prophylaxis.

As shown in Figure 2, according to adherence assessments by pill count and questionnaires at each visit, the proportion of adherence greater than 90% was lower and the rate of switching from event-driven to daily regimen was higher for GSN apps’ users than nonusers. As shown in Table 2, in the multivariable analysis of generalized estimation equation with logistic regression, GSN apps’ use was also found to be a significantly independent predictor of adherence over 90% with adjusted odds ratio (aOR) of 0.8 (95% CI 0.6-1.0; *P=*.037). In terms of the medication regimen, the adherence to the daily regimen was higher than that to the event-driven regimen (aOR 3.8, 95% CI 3.1-4.6). The subgroup analysis of different regimens showed that the adherence of GSN apps’ users to the event-driven PrEP was lower than that of nonusers (aOR 0.7, 95% CI 0.6-0.9). Other independent predictors of adherence included age (vs 18-24 years; 25-34: aOR 1.3, 95% CI 1.0-1.7; 35-65 years: aOR 1.4, 95% CI 1.0-1.8), college education and above (aOR 1.3, 95% CI 1.0-1.7), daily regimen (vs event-driven regimen; aOR 3.8, 95% CI 3.1-4.6), having multiple male sex partners (vs 0-1 male sex partners; 2-3: aOR 1.2, 95% CI 1.0-1.5; ≥4: aOR 1.3, 95% CI 1.1-1.7), multiple casual male sex partners (vs 0; 1-3: aOR 1.1, 95% CI 0.9-1.3; ≥4: aOR 1.3, 95% CI 1.0-1.6), number of CAI instances of 3 or more (vs no CAI; aOR 1.2, 95% CI 1.0-1.5), and substance use (vs no substance use; aOR 0.7, 95% CI 0.5-0.9; Table 2).

For self-reported adherence measures, GSN apps’ users showed a statistically marginally lower level of adherence than nonusers in the event-driven regimen (aOR 0.7, 95% CI 0.4-1.0, *P*=.060). PrEP coverage of last sex events was significantly lower with GSN apps’ users than with nonusers on the event-driven regimen (aOR 0.6, 95% CI 0.4-1.0, *P*=.038; Table 3).

As much as 9.7% (96/993) of PrEP users switched regimens, including 82 who switched once and 14 who switched more than once. Of the 111 regimen-switching events, 67 (60.4%) were from event-driven to the daily regimen, and 44 (39.6%) were from event-driven to the daily regimen. Interestingly, based on the above multivariable analysis, GSN apps’ users were more likely to switch regimens from event-driven to daily use, compared with nonusers (aOR 1.8, 95% CI 0.9-3.3, *P*=.084; Table 3).

Additionally, 4.40% (45/1023) of participants discontinued PrEP and dropped out of the study, and there were no significant differences in rates of discontinuation between GSN apps’ users and nonusers (4.4% [23/520] vs 4.4% [22/503], *P*=.796).

**Table 2:** Generalized estimated equation analysis of behaviors during follow-up as predictors of over 90% adherence to pre-exposure prophylaxis by pill counting (N=993).^a^

Factors			Total, n (%)	Adherence >90%, n (%)		Multivariate analysis		
						aOR^b^ (95% CI)		*P*-value
**Age (years)**								
	18-24		230 (23.2)	146 (63.5)		Reference		
	25-34		501 (50.5)	333 (66.5)		1.3 (1.0 to 1.7)		.028
	35-65		262 (26.4)	179 (68.3)		1.3 (1.0 to 1.8)		.036
**Education**								
	High school or below		175 (17.6)	113 (64.6)		Reference		
	College and above		818 (82.4)	545 (66.6)		1.3 (1.0 to 1.7)		.037
**Sexual role with male**								
	Oral		57 (5.7)	30 (52.6)		Reference		
	Top		341 (34.3)	233 (68.3)		2.0 (1.4 to 2.8)		<.001
	Bottom		279 (28.1)	193 (69.2)		2.1 (1.5 to 3.0)		<.001
	Versatile		316 (31.8)	202 (63.9)		1.8 (1.2 to 2.6)		.002
**Main venue to seek male sex partners**								
	Others		256 (25.8)	181 (70.7)		Reference		
	GSN apps^c^		737 (74.2)	477 (64.7)		0.8 (0.6 to 1.0)		.037
**Sexual behaviors in past 3 months**								
	**Number of male sex partners**							
		0-1	303 (30.5)	188 (62.0)		Reference		
		2-3	336 (33.8)	237 (70.5)		1.2 (1.0 to 1.5)		.050
		≥4	354 (35.6)	233 (65.8)		1.3 (1.1 to 1.7)		.012
	**Number of male casual sex partners**							
		0	474 (47.7)	309 (65.2)		Reference		
		1-3	283 (28.5)	198 (70.0)		1.1 (0.9 to 1.3)		.289
		≥4	236 (23.8)	151 (64.0)		1.3 (1.0 to 1.6)		.054
	**Number of CAI^d^**							
		0	522 (52.6)	343 (65.7)		Reference		
		1-2	135 (13.6)	85 (63.0)		1.0 (0.8 to 1.2)		.816
		≥3	336 (33.8)	230 (68.5)		1.2 (1.0 to 1.5)		.041
**Substance use**								
		No	559 (56.3)	514 (91.9)		Reference		
		Yes	434 (43.7)	401 (92.4)		0.7 (0.5 to 0.9)		.014
**PrEP^e^ dosing regimens**								
	Event-driven regimen		466 (46.9)	245 (52.6)		Reference		
	Daily regimen		527 (53.1)	413 (78.4)		3.8 (3.1 to 4.6)		<.001

^a^All estimates were from generalized estimating equation logistic models, adjusted for ethnic, local residents, and annual income.

^b^aOR: adjusted odds ratio.

^c^GSN: geosocial networking.

^d^CAI: condomless anal intercourse.

^e^PrEP: pre-exposure prophylaxis.

**Table 3:** Adherence to pre-exposure prophylaxis and change of regimen among men who have sex with men that are geosocial networking app users and nonusers (N=993).^a^

	Total (GSN apps’^b^ user vs nonuser)		Daily regimen (GSN apps’ user vs nonuser)			Event-driven regimen (GSN apps’ user vs nonuser)		
	aOR^c^ (95% CI)	*P*-value		aOR (95% CI)	*P*-value		aOR (95% CI)	*P*-value
Pill counting (vs adherence ≤90%)	0.8 (0.6 to 1.0)	.037		0.8 (0.6 to 1.1)	.205		0.7 (0.6 to 0.9)	.019
Self-report pills used as recommended (vs No)	0.9 (0.7 to 1.1)	.224		1.0 (0.7 to 1.3)	.968		0.7 (0.4 to 1.0)	.060
Self-report last sex events fully covered by PrEP^d^ (vs No)	0.7 (0.5 to 1.0)	.057		1.1 (0.6 to 2.0)	.869		0.6 (0.4 to 1.0)	.038
Ever switched regimens (vs continuing initial regimen)	1.5 (0.9 to 2.6)	.161		1.7 (0.9 to 3.3)	.104		1.2 (0.5 to 2.6)	.681
Event-driven to daily PrEP	1.8 (0.9 to 3.3)	.084		1.7 (0.9 to 3.3)	.104		—	—
Daily to event-driven PrEP	1.2 (0.6 to 2.6)	.633		—	—		1.2 (.5 to 2.9)	.681

^a^All estimates are from generalized estimating equation logistic models, adjusted for ethnic, local residents, and annual income.

^b^GSN: geosocial networking.

^c^aOR: adjusted odds ratio.

^d^PrEP: pre-exposure prophylaxis.

## Discussion

### Principal Findings

These study results represented, to our best knowledge, the first evidence on the correlation between GSN apps’ use and PrEP adherence in a real-world setting with options for daily or event-driven regimens to address bottlenecks in order to optimize PrEP adherence. Our results revealed that a high proportion of PrEP users mainly seek sexual partners through GSN apps, and GSN apps’ users are at a higher sexual risk than other PrEP users. Worryingly, GSN apps’ users had lower levels of medication adherence and higher rates of regimen switching during follow-up than GSN apps’ nonusers. These data suggest the importance of reaching MSM at high risk through GSN platforms and providing them with education and interventions to improve PrEP adherence.

In this study, nearly three-quarters of MSM on PrEP reported seeking sexual partners mainly via GSN apps at baseline, which was higher than those reported in the general MSM population (56.0%) [2]. A study on American MSM found that those using GSN apps seemed more likely to start PrEP (aOR 1.6) compared with non-GSN apps’ users, which seems to partly explain the high level of GSN apps’ usage among MSM on PrEP [15]. Additionally, our results are similar to those from previous studies that found a higher proportion of HIV-related high-risk sexual behaviors among GSN apps’ users compared to nonusers [4,7,24,25]. GSN apps offer many advantages not available with other traditional ways of encountering a sexual partner and making it easier to locate and screen via the app itself [4,7,24].

Medication adherence and regimen switching are important factors affecting the effectiveness of PrEP; however, there has been a lack of evidence on the effect of GSN apps’ use on the above outcome variables. Our results found that although GSN apps’ users were at a higher sexual risk than non-GSN apps’ users, they had lower level of medication adherence to PrEP, and higher rate of regimen switches during follow-up. The observed decrease in PrEP adherence among GSN apps’ users could be attributed to their higher frequent substance using behaviors than nonusers. Previous research had demonstrated that substance use with sex is strongly associated with poor adherence to HIV treatments [26]. Additionally, this study observed a potential decline in the adherence of GSN users during the 6-12-month study period, which may be explained by the higher proportion of younger MSM among GSN users compared with nonusers. Our results also found a lower PrEP coverage of sex events among GSN apps’ users compared with nonusers to further support this hypothesis. Furthermore, this study found that GSN apps’ users were prone to transition from event-driven PrEP to daily dosing regimen than nonusers, although there was no statistically significant difference between the 2 groups in the choice of regimen at initiation. Additional work is needed to address the high rate of regimen switching among GSN apps’ users, and response recommendations should be added to the WHO and country PrEP guidelines that recommend MSM currently using GSN apps to choose a more appropriate daily regimen based on their active sexual behavior. Daily dosing regimens are recommended by guidelines and some trial evidence for MSM with more frequent and irregular sexual behaviors due to the regimen’s simplicity and ease of operation [17,27,28]. Given that GSN apps’ users have a high conversion rate for medication regimens, they should be provided with timely education on new medication regimens, not only when PrEP is initiated, but also when they switch regimens. Because of the low dropout rate in this study, no significant difference was found in the termination rate of PrEP between GSN users and nonusers, and the outcome remains to be further observed and confirmed in a larger population with low cohort retention rate in the future.

Given that the GSN platforms cover most MSM using PrEP with high risk levels but low adherence, adherence intervention through eHealth on the GSN platforms may be an ideal way to optimize adherence in the large-scale implementation of PrEP. The soaring number of dating app users offers unique opportunities to reach out to at-risk MSM for a broad range of health education and interventions [20,29]. GSN app-based advocacy has proven effective in expanding HIV testing and prevention interventions recruitment [30-35]. However, how to effectively deliver eHealth of PrEP adherence on GSN apps needs to be further explored [36]. Although banner ads in apps may be expensive [29,37,38], they can be used to effectively educate the public about PrEP and raise awareness, such as about different PrEP regimens. Pop-up ads in apps appear to be suitable for use as a just-in-time adherence intervention that can be set up on the dating apps to improve PrEP medication adherence [39]. A study conducted in the United States suggests that detailed user profiles on social platforms could help increase sexual health awareness of MSM [40]. This experience could be extended to reduce HIV high-risk behaviors of PrEP users by adding this setting to the GSN platform in China and other countries with similar HIV rates. Offline adherence support also must provide specific assistance to MSM with GSN apps’ usage by providing counseling on medication regimens and switching regimens during PrEP initiation to minimize regimen switching.

### Strengths and Limitations

A major strength of this study was the assessment of adherence and regimen switches among MSM who reported using GSN apps, which increased our awareness of the correlation between GSN apps’ use and adherence to PrEP. Furthermore, this study was based on a national multicenter real-world study that simulated real-world scenarios in which participants were able to choose their regimens and to switch between daily and event-driven dosing regimens if desired. However, there were also several limitations in this study. The follow-up time was relatively short, and long-term outcomes may not be observed. Medication adherence may be overestimated to some extent due to the free availability of the PrEP and certain incentives in this demonstration study. This study did not further analyze the effect of GSN apps on HIV-prevention efficacy and drug concentration for PrEP, which future studies should examine. Although this study tries to simulate real-world scenarios as much as possible, compliance may be overestimated to some extent due to the free drug supply and the limited amount of incentive costs used to motivate PrEP use, as PrEP is not yet available in China. This study used a variety of methods to supervise the return of medicines, and pill counting remains inevitably affected by uncontrollable reasons such as forgetting to take them. Therefore, our study also uses questionnaires to comprehensively evaluate PrEP adherence from multiple perspectives. Although this study used flexible online and offline recruitment methods to reach all levels of MSM, similar to previous studies, the very high proportion of MSM with university degrees who start PrEP may not be representative of the entire MSM population in China. Therefore, further research is necessary to examine the causality between app use and the PrEP adherence of MSM.

### Conclusions

This study found that GSN apps’ users who account for a large proportion of daily and event-driven oral PrEP users have lower adherence and are more likely to switch regimens despite their higher sexual risk than GSN apps’ nonusers. Based on these results, GSN platforms provide a unique opportunity to reach high-risk MSM for promoting PrEP outreach, and it is also necessary to customize medication adherence interventions for GSN apps’ users to improve the preventive effect of PrEP.

## Abbreviations

aOR: adjusted odds ratio
CAI: condomless anal intercourse
CASI: computer-assisted self-interview
GSN: geosocial networking
MSM: men who have sex with men
PrEP: pre-exposure prophylaxis
STI: sexually transmitted infection
TDF/FTC: tenofovir; disoproxil fumarate plus emtricitabine
