# Correction: Risk-Taking Behaviors and Adherence to HIV Pre-Exposure Prophylaxis in Users of Geosocial Networking Apps: Real-World, Multicenter Study

**DOI:** 10.2196/25565

**Published:** 2020-11-09

**Authors:** Hongyi Wang, Jing Zhang, Zhenxing Chu, Qinghai Hu, Willa Dong, Xiaojie Huang, Yaokai Chen, Hui Wang, Xiaoqing He, Lukun Zhang, Zhili Hu, Rantong Bao, Shangcao Li, Hang Li, Sitong Cui, Xia Jin, Haibo Ding, Wenqing Geng, Yongjun Jiang, Junjie Xu, Hong Shang

**Affiliations:** 1 NHC Key Laboratory of AIDS Immunology (China Medical University), National Clinical Research Center for Laboratory Medicine, The First Affiliated Hospital of China Medical University Shenyang, Liaoning Province China; 2 Key Laboratory of AIDS Immunology, Chinese Academy of Medical Sciences Shenyang China; 3 Key Laboratory of AIDS Immunology of Liaoning Province Shenyang China; 4 Collaborative Innovation Center for Diagnosis and Treatment of Infectious Diseases Hangzhou China; 5 Department of Health Behavior, Gillings School of Global Public Health, The University of North Carolina at Chapel Hill Chapel Hill, NC United States; 6 Center for Infectious Diseases, Beijing Youan Hospital, Capital Medical University Beijing China; 7 Chongqing Public Health Medical Center Chongqing China; 8 Shenzhen Third People’s Hospital Shenzhen China

In “Risk-Taking Behaviors and Adherence to HIV Pre-Exposure Prophylaxis in Users of Geosocial Networking Apps: Real-World, Multicenter Study” (J Med Internet Res 2020;22(10):e22388) the authors noted one error.

A typographical error in the originally published version of the paper rendered the second half of the article title as:

Real-Word, Multicenter Study

This has been corrected to:

Real-World, Multicenter Study

The correction will appear in the online version of the paper on the JMIR Publications website on November 9, 2020, together with the publication of this correction notice. Because this was made after submission to PubMed, PubMed Central, and other full-text repositories, the corrected article has also been resubmitted to those repositories.

